# Linguistic characteristics of patients with bulimic symptomatology in an online post-treatment program: an exploratory study

**DOI:** 10.1007/s40519-014-0136-1

**Published:** 2014-06-25

**Authors:** Ágnes Mezei, Hayriye Gulec, Edit Czeglédi, Adorján Fritz, Ferenc Túry

**Affiliations:** Institute of Behavioural Sciences, Semmelweis University, Nagyvárad tér 4, Budapest, 1089 Hungary

**Keywords:** New technologies, E-mental health, Bulimia nervosa, Group chat, Linguistic analysis

## Abstract

**Background:**

No former investigation has been performed related to the linguistic characteristics of patients with eating disorders using online synchronous communication mediums like chats.

**Objective:**

The purpose of this study was to investigate the linguistic predictors of improvement in eating disorder-related attitudes, behaviours and emotional distress of patients with eating disorders.

**Methods:**

Thirty-nine women, who had received treatment for bulimia nervosa or related eating disorders not otherwise specified, utilized the moderated therapeutic group chats of an Internet-based program for 4 months. The main themes of 134 session transcripts were created using a general inductive approach. The frequency of dictionary words in the text corpus was processed by the NooJ linguistic software. Eating Disorder Examination Questionnaire and Depression Anxiety Stress Scale were administered at the beginning and at the end of 4 months. No valid questionnaire data could be obtained from 11 participants, so the statistical analyses were performed in a sample of 28 subjects.

**Results:**

According to the results of multiple linear regression analyses, higher ratio of words related to “family of origin” was associated with improvements in eating disorder-related attitudes, emotional distress, and reduction in the frequency of binge eating episodes (β = 0.73, *p* < 0.001; β = 0.67, *p* = 0.002; β = 0.53, *p* = 0.039, respectively).

**Discussion:**

The expression of “family of origin” words following treatment termination was a linguistic predictor of improvement during group chat communication of patients with bulimic symptomatology. The results show the importance of family issues in enhancing the treatment outcome and provide preliminary evidence to address this topic during online chat moderation.

## Introduction

The new communication technologies have changed the medicine, and a new subdiscipline emerged. E-health is the use of modern communication technologies in healthcare delivery with a role in prevention, diagnostics, therapy, rehabilitation and health education of several disorders. There is accumulating evidence indicating usefulness of these new methods for patients with bulimia nervosa (BN), binge eating disorder, and eating disorders not otherwise specified (EDNOS) [1–3]. Over the past decade an emerging direction in e-health has been the evaluation of the linguistic characteristics of patients using various Internet platforms, such as forums, support groups, chats and e-mails. Research in this field has investigated, e.g., patients with cancer [4–7], and conditions like emotional, personality, or behavioural disorders [8, 9]. Few studies investigated the online linguistic characteristics of patients with eating disorders within the context of Internet pro-recovery discussion groups [10], pro-anorexia (“pro-ana”) and pro-recovery message boards and web pages [11], pro-ana social networking sites [12], and pro-ana online discussion forums [13]. However, the knowledge on the use of language among patients with eating disorders utilizing interactive mediums such as Internet chat rooms is not existent.

Communication in Internet chat rooms is generally text-based and the text correspondence resembles face-to-face interactions as both of them take place in real time. However, features such as invisibility and lack of visual cues in Internet chat rooms are discussed to bring about faster and greater openness compared to traditional therapy settings, also referred to as “online disinhibition effect” [14]. In addition, in case of eating disorders, the invisibility of the body could allow different aspects of therapeutic relationship to appear compared to face-to-face settings, where the body is approachable [15]. Given the increasing use of e-health interventions in healthcare delivery of patients with eating disorders, knowledge on these processes may provide valuable information into the poorly understood process and course of eating disorders and inform about the best practice approaches in such interventions. For this purpose, in an exploratory study we investigated the online written language of patients with bulimic symptomatology within moderated therapeutic group chats of an Internet-based support program and investigated linguistic predictors of improvement in eating disorder-related attitudes, behaviours and emotional distress. To our knowledge, this is the first study to conduct linguistic analysis of text written during therapeutic group chat sessions of patients with eating disorders.

## Methods

### Participants and recruitment

Participants who were studied as part of an online support program (EDINA) within a European collaboration constituted the sample of the current study [16]. The inclusion criteria were as follows:

- females aged ≥16 years, treated for BN, binge eating disorder, EDNOS, or anorexia nervosa binge-purging subtype during the previous year,
- body mass index (BMI) ≥17.5,
- weekly Internet access,

The diagnoses were based on the DSM-IV [17].

The exclusion criteria were major organic and substance-induced disorders, comorbid psychotic disorders, suicidal risk, and insufficient knowledge of Hungarian language.

The treatment modalities used in the therapy of the participants were cognitive-behaviourally oriented inpatient treatment in a psychosomatic department; outpatient group CBT; psychodynamically oriented therapy; family therapy; pharmacotherapy and supportive therapy; and mostly eclectic therapy using several elements from different treatment approaches. Due to this diversity, a distinct variable of former treatment modalities could not be created.

In the present study, the sample consisted of 39 participants who utilized the group chat component of the program for 4 months and provided available questionnaire data. Data were collected between February 2010 and June 2012. All participants provided informed consent before study inclusion and parental consent was obtained when participants were younger than 18 years. The study was approved by the Institutional Research Ethics Board of the Semmelweis University, Budapest.

### Measures

Participants completed self-report online questionnaires at baseline which assessed sociodemographic variables with respect to age, years of education, marital status, anthropometric data (i.e., body weight and height), duration of illness, type of the last treatment received relating to eating disorder problems and whether multiple treatment histories existed. They were also assessed on eating disorder-related features and emotional distress at baseline and at the end of the 4 months. The core eating disorder symptoms and attitudes were assessed by the Eating Disorder Examination Questionnaire (EDE-Q; [18]). For the current study, EDE-Q was translated into Hungarian language, edited and translated back by a bilingual translator. Both versions were reviewed by the study team, and if necessary, adjustments were made. Internal consistencies of the total and subscale scores were acceptable (Cronbach’s alphas range from 0.66 to 0.94). Emotional distress was assessed by the Depression Anxiety Stress Scale (DASS-21; [19]). The DASS-21 consists of a total and three subscale scores (i.e., depression, anxiety and stress). For the current study, the Hungarian version of DASS-21 suggested by the DASS working group [20] was adopted and yielded good internal consistencies (Cronbach’s alphas range from 0.77 to 0.94).

### The online intervention

The EDINA offers participants an information and communication platform for peer support and professional counselling, and was described elsewhere [16]. The modules of the program include online information material, a forum, online symptom monitoring and supportive feedback system and chats (group and individual). The 90–min group chat sessions took place at the password protected chat room of the program at a fixed time every week and were moderated by a trained therapist (A.M.), who received supervision from a senior clinician and researcher in eating disorders (F.T.). The groups were open, anonymous and generally consisted of 5–8 participants. The group chats focused on “here and now” topics and aimed at providing professional support and the opportunity to meet virtually with other people who suffer or suffered from the same problem. Each session was stored by the underlying software Web-Akquasi [21].

### Content analysis

The chat scripts were extracted and the typos and grammatical mistakes were corrected by the study team before conducting the content analysis. In the extracted documents each line belonged to one participant or the online counsellor, and was indicated by the time of sending the message (hh:mm:ss) and the pseudonym. In total, transcripts of 134 chat sessions from 39 participants in Hungarian were available during the study period. The mean group size was 8.2 (SD = 2.15; range 3–14). The participants who wrote <50 words in a group chat session or did not have available data at the beginning or at the end of 4-month chat period were excluded (*N* = 11; 28 %). The 4-month-long chat corpus was created by compiling four monthly text corpora which contained four group chat sessions each. The final chat corpus consisted of 135,805 words. On average, participants wrote 3,482.2 words (SD = 2,551.83; range 326–8,783) during 4 months. For calculating the absolute and relative occurrence of the words in the text corpus, NooJ linguistic software was used [22]. Relevant themes of the chat scripts were identified based on a general inductive approach [23]. First, the chat scripts were overviewed with respect to the main themes of the text, and then the central themes (i.e., the themes of the word categories used in the study) were subsequently created for the description of the online content. The central word categories contained the most frequently used topics from well-known questionnaires of eating disorder and body image disorder-related characteristics (for review: [24]). Six trained and independent judgers classified each word into these categories to create the corpus-based dictionary of the study. If four of the six judgers (2/3) classified a word into one of these categories, the place of the word was finalized. Different forms of the words were retained in the dictionary (e.g., plural, tenses, and cases). The following nine word categories were formed: body and clothing (643 words: e.g., body shape, face, bottom), expressed negative emotions (25 words: e.g., hate, anger), family of origin (269 words: e.g., grandparents, parents, siblings), food and eating (762 words: e.g., kcal, apple, chocolate), illness, symptoms and therapy (876 words: e.g., ill, anorexia, depression, treatment), important others (241 words: relatives, friends, children, husband), physical aggression (57 words: e.g., beat, hit, kick), sexuality and intimacy (137 words: e.g., love, desire, sex), sports and physical activity (139 words: e.g., bicycling, training, running). In addition, we included first-person pronouns (34 words: e.g., myself, for me, with me, with us) to assess the predictive value of self-reference on assessed dimensions. To determine the monthly ratio of each word category, the frequency of words was divided by the total number of words in the monthly corpus for each participant. Then, the mean of the monthly ratios relating to each word category was calculated for the 4-month study period. Further statistical analyses were carried out based on these indices.

### Statistical analysis

The internal consistencies of the questionnaires were estimated using the Cronbach’s alphas. For continuous variables paired samples *t* test was computed to compare the scores at baseline and the end of 4 months, and the effect sizes were calculated using the Cohen’s *d* [25]. A composite score of purge behaviours was calculated by adding the number of vomit episodes and laxative misuse episodes. Changes in eating disorder attitudes and behaviours (EDE-Q), and in emotional distress (DASS-21) were calculated subtracting the values at the end of 4 months from the baseline values, where higher change scores indicated more improvement. Change scores of cognitive and behavioural symptoms of eating disorders and emotional distress were correlated with the average ratio of the word categories in the chat corpus. In case of non-normal distribution, Spearman’s rank correlation was used. Based on the significant correlations between the change scores and word categories, multiple linear regression analyses were conducted adjusting for potential confounding variables. In case of non-normal distribution, Spearman’s partial rank correlation coefficient was calculated. We could not collect valid questionnaire data from 11 participants, so the analyses were performed in a sample of 28 subjects. No significant differences were found between the study group and the excluded participants in terms of sociodemographic variables (age, marital status, years of education), height, duration of illness, and former treatments. However, the body weight of the subjects who refused completing questionnaires was lower at a tendency level (*t*_(35)_ = −1.730, *p* = 0.092, Cohen’s *d* = 0.68), and the BMI of these subjects was significantly lower (*t*_(35)_ = −2.040, *p* = 0.049, Cohen’s *d* = 0.80) in comparison to the participants who completed the questionnaires.

## Results

Respondents (*n* = 28) were on average 27.5 (SD = 6.36, range 19–49) years old and had a mean education duration of 14.7 (SD = 2.40, range 10–20) years. Fifty-seven percent of the participants reported to be married and 43 % had divorced. For 11 participants (39 %), the illness duration was longer than 5 years. Eleven participants (39 %) reported receiving multiple eating disorder-related treatments before the study inclusion. Relating to the last treatment, 11 % (*n* = 3) of the participants were inpatients, and 89 % were outpatients (self-help group: *n* = 7, counselling: *n* = 2, psychotherapy: *n* = 15, other treatment: *n* = 1). There were no significant differences between the baseline and end of 4-month scores with respect to eating disorder-related behaviours and attitudes, emotional distress and BMI (Table 1).

**Table 1:** Comparisons of baseline and end of 4-month scores of eating disorder-related behaviours and attitudes, emotional distress, and BMI

Variables	Baseline *M* (SD)	End of 4 months *M* (SD)	*t* (*p*)	Cohen’s *d*
BMI	21.33 (4.23)	21.50 (4.01)	−0.562 (*p* = 0.579)	−0.15
EDE-Q				
Restraint	2.86 (1.70)	2.40 (1.91)	1.654 (*p* = 0.110)	0.45
Eating concerns	2.21 (1.40)	1.97 (1.30)	0.959 (*p* = 0.346)	0.26
Weight concerns	3.27 (1.25)	2.89 (1.50)	1.167 (*p* = 0.253)	0.31
Shape concerns	3.78 (1.41)	3.39 (1.52)	1.161 (*p* = 0.256)	0.31
Total	3.03 (1.25)	2.66 (1.38)	1.470 (*p* = 0.153)	0.39
Binge eating episodes	8.00 (11.34)	7.36 (9.67)	0.609 (*p* = 0.548)	0.17
Purge episodes	9.57 (15.49)	7.86 (13.12)	1.42 (*p* = 0.168)	0.41
DASS-21				
Depression	15.20 (11.03)	16.32 (11.64)	−0.443 (*p* = 0.662)	−0.13
Anxiety	10.72 (9.45)	9.20 (8.60)	0.967 (*p* = 0.343)	0.27
Stress	17.04 (7.83)	18.24 (8.74)	−0.573 (*p* = 0.572)	−0.16
Total	42.96 (25.89)	43.76 (25.82)	−0.146 (*p* = 0.885)	−0.04

*n* = 25–28

*EDE-Q* Eating Disorder Examination Questionnaire, *DASS-21* Depression Anxiety Stress Scale

The total ratio of the word categories constituted 5.5 % (7,460 words) of the total text produced by the participants during the 4-month group chat attendance. It is usual that the majority of a text cannot be assigned to any category [23, 26]. In a similar study by Wolf, Chung and Kordy [9], the ratio of the dictionary nouns constituted 3 % of the total words and they argued that this percentage is acceptable as around 50 % of the words used in natural languages consists of particles. They referred to the complexity of the language as a human behaviour, and if we extract patterns from it, simplifications are necessary on the conceptual and operational level. In addition, Chung and Pennebaker [26] emphasized that the communicational value of the text depends on the functional words (or “junk” words, particles) as well, which serve as the cement that holds the content words together. However, in this pilot study we aimed at the evaluation of content words (i.e., nouns, verbs and adjectives).

**Fig. 1:**
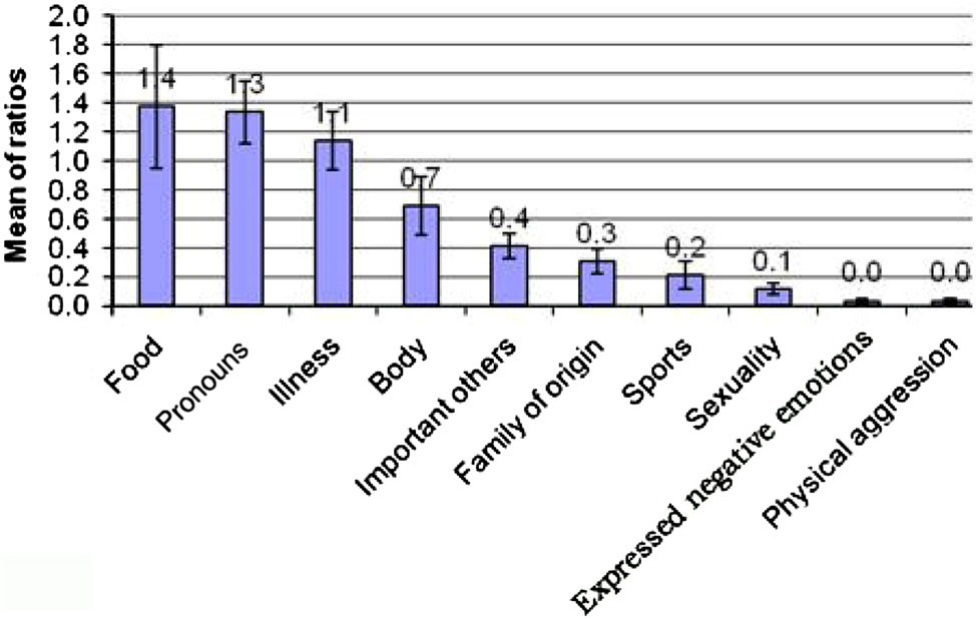
The mean ratios of the word categories in the chat corpus during the 4-month period. 95 % confidence intervals of means are represented

The mean ratio of the word categories in the corpus are represented in Fig. 1.

During the 4-month period, the mean reduction in binge eating episodes was 0.64 (SD = 5.59, range −11 to 9) and the mean reduction in purging episodes was 1.71 (SD = 6.40, length: −16 to 19). According to the correlational analyses, family of origin word category demonstrated significant positive moderate to strong associations with most of the variables except change in purge behaviours, where an association was found at a tendency level. The word category of important others showed significant positive moderate correlation with the change in purge behaviours. In addition, significant positive moderate to strong relationships were found between the expressed negative emotions word category and change in eating concerns and weight concerns scales of the EDE-Q (Table 2).

**Table 2:** Correlations between the word categories and the change relating to eating disorder-related behaviours, attitudes and emotional distress

Change scores	Physical aggression	Illness	Family of origin	Expressed negative emotions	Food	Important others	Sport	Sex	Body	First-person pronouns
EDE-Q										
Restraint	−0.18	0.07	0.50**	0.12	0.00	0.34^+^	−0.02	−0.11	0.08	0.08
Eating concerns	−0.08	−0.08	0.65***	0.40*	−0.10	0.34^+^	0.05	0.18	0.12	0.03
Weight concerns	0.05	−0.04	0.53**	0.46*	−0.15	0.07	−0.05	0.14	0.02	−0.03
Shape concerns	0.04	−0.03	0.52**	0.33^+^	−0.03	0.15	0.14	0.00	0.20	0.09
Total	−0.04	−0.02	0.65***	0.39*	−0.08	0.25	0.04	0.06	0.13	0.05
Frequency of binge eating episodes	−0.05	−0.25	0.54**	0.33^+^	−0.07	0.21	0.20	0.14	0.19	0.03
Frequency of purge episodes	−*0.19*	−*0.26*	*0.34* ^+^	*0.20*	*0.14*	0.41*	*0.20*	*0.17*	*0.14*	−*0.28*
DASS-21										
Depression	−0.05	−0.02	0.51**	0.36^+^	−0.17	−0.07	0.28	−0.08	0.25	−0.13
Anxiety	−0.20	−0.02	0.59**	0.17	−0.09	0.15	−0.02	−0.06	0.01	−0.23
Stress	−0.26	−0.06	0.57**	0.27	−0.01	−0.02	0.07	0.08	0.29	−0.29
Total	−0.18	−0.04	0.62**	0.32	−0.11	0.00	0.15	−0.02	0.23	−0.24

*n* = 25–28. Italic type means Spearman’s rank correlation coefficient

*EDE-Q* Eating Disorder Examination Questionnaire, *DASS-21* Depression Anxiety Stress Scale

^+^ *p* < 0.10, * *p* < 0.05, ** *p* < 0.01, *** *p* < 0.001

Based on the results of the correlational analyses, the relevant word categories were tested in multivariate models adjusting for age, baseline BMI, years of education, chronicity, baseline frequency of binge eating and purging behaviours, baseline eating disorder-related cognitive attitudes (EDE-Q total score), and baseline emotional distress (DASS-21 total score) (Table 3). According to the results of the multiple linear regression analyses, higher ratio of words related to family of origin (β = 0.73, *p* < 0.001), higher baseline BMI (β = 0.46, *p* = 0.003), lower baseline frequency of binge eating episodes (β = −0.42, *p* = 0.025), and lower baseline emotional distress (DASS-21 total score; β = −0.45, *p* = 0.022) were significantly associated with improvements in eating disorder-related attitudes assessed by the EDE-Q total score at the end of 4 months. In addition to that, higher ratio of words related to expressed negative emotions (β = 0.24, *p* = 0.088) and higher baseline EDE-Q total score (β = 0.37, *p* = 0.068) were associated with improvements in eating disorder-related attitudes at a tendency level. The model explained 70.7 % of the variance. With regards to eating disorder-related behaviours, higher ratio of words related to family of origin (β = 0.53, *p* = 0.039) was significantly associated with reduction in the frequency of binge eating episodes. The model explained 28.2 % of the variance. According to the result of Spearman’s partial rank correlational analysis, using higher rate of words related to important others showed marginally significant association with reduction in purge behaviours (Spearman *r*_p_ = 0.42, *p* = 0.065). Lastly, using higher ratio of words related to family of origin (β = 0.67, *p* = 0.002) and longer duration of illness (β = 0.45, *p* = 0.046) were significantly associated with improvements in emotional distress assessed by the DASS-21 total score at the end of 4 months. Higher BMI at baseline showed relationship with improvements in emotional distress at a tendency level (β = 0.29, *p* = 0.070). The model explained 59.7 % of the variance.

**Table 3:** Results of the multiple linear regression analyses

Variables	Improvement in eating disorder-related cognitions^a^			Improvement in frequency of binge eating episodes^b^			Improvement in emotional distress^c^		
	Beta	*t*	Sig.	Beta	*t*	Sig.	Beta	*t*	Sig.
Family of origin	0.73***	4.889	<0.001	0.53*	2.260	0.039	0.67**	3.846	0.002
Expressed negative emotions	0.24^+^	1.834	0.088	–	–	–	–	–	–
Age	0.22	1.385	0.188	0.25	1.008	0.330	−0.15	−0.795	0.439
BMI at baseline	0.46**	3.625	0.003	0.20	1.017	0.325	0.29^+^	1.949	0.070
Years of education	0.16	1.012	0.329	0.25	1.044	0.313	0.16	0.876	0.395
Chronicity	−0.01	−0.029	0.977	−0.22	−0.801	0.435	0.45*	2.174	0.046
Binge eating episodes at baseline	−0.42*	−2.512	0.025	0.27	1.007	0.330	−0.28	−1.445	0.169
Purge episodes at baseline	0.18	1.022	0.324	0.07	0.243	0.811	0.23	1.134	0.275
EDE-Q total score at baseline	0.37^+^	1.979	0.068	0.20	0.712	0.487	−0.15	−0.699	0.495
DASS-21 total score at baseline	−0.45*	−2.582	0.022	−0.22	−0.792	0.440	0.34	1.654	0.119
Adjusted *R* ^2^	70.7 %			28.2 %			59.7 %		

*n* = 25

^a^Based on change in Eating Disorder Examination Questionnaire (EDE-Q) total score

^b^Based on change in frequency of binge eating assessed by the EDE-Q

^c^Based on change in Depression Anxiety Stress Scale (DASS-21) total score

^+^ *p* < 0.10, * *p* < 0.05, ** *p* < 0.01, *** *p* < 0.001

## Discussion

In the current study, we investigated the linguistic characteristics of patients with eating disorders who had treatment for BN or related symptomatology and utilized the weekly moderated group chat sessions of an Internet-delivered support program (EDINA; [16]) following treatment termination for 4 months. To the best of our knowledge, this was the first study to analyze a synchronous online communication medium in patients with eating disorders. The present study consistently showed that using higher ratio of words related to family of origin (i.e., grandparents, parents, siblings) during chat sessions was associated with improvements in eating disorder-related attitudes, frequency of binge eating episodes and emotional distress from baseline to end of 4 months. These results are remarkable, highlight the importance of family issues in enhancing the treatment outcome and provide preliminary evidence to address this topic during online chat moderation of patients with bulimic symptomatology at post-treatment.

Considering the influence of the moderator on the topics discussed during the chat, no major effect can be assumed. The online intervention was moderated by a clinical psychologist, who is trained in psychodynamic, cognitive-behavioural and family therapeutic orientation. However, the style of the chat moderation was neutral and reactive. The moderator asked open questions at the beginning of each session (e.g.: “Is there anything you would like to talk about today?”), stimulated interaction between the participants and used positive reframing and emotional support throughout the chat sessions.

As our data demonstrate, family issues seem to be important in the aftercare of eating disordered patients. Although the context of the chat is aftercare, these results may have some therapeutic implications. If a new therapy is needed (e.g., after a relapse), family-based therapy, individual systemic therapy, interpersonal therapy, adolescent-focused psychotherapy, or other therapeutic modalities with a focus on family issues could be preferred. Obviously, the importance of words relating to family members can be the consequence of either the helping attitude of the family members or the family stress. We relied on the frequency of words in each word category for the analyses. Thus, we cannot speculate on the context within which they were produced.

The linear association between the frequent use of words relating to family of origin and improvements in purge behaviours at a tendency level, and the significant association between the frequent use of words relating to important others (e.g., relatives, friends, children, husband) and improvements in purge behaviours may reflect that these symptoms have often strong interpersonal message, e.g., the purging symptoms may serve as regulating factors in intimacy [27, 28]. Dealing with fear of intimacy in an anonymous environment through writing own emotions and “listening” to the others’ could have improved awareness on emotional conflicts and contributed to the development of healthier coping mechanisms.

We found, at a tendency level, a linear association between using higher ratio of words relating to expressed negative emotions (e.g., hate, anger) and improvements in eating disorder-related attitudes. Studies showed positive psychological and somatic effects of writing about emotions in different populations [29–31]. After a disclosure writing intervention, it was found that participants who wrote a high number of positive emotion words, a moderate number of negative emotion words, and an increasing number of cognitive (i.e., causal and insight) words related to traumas over 3 days were more likely to benefit from the intervention [32]. In the current study, we did not investigate the use of emotion and cognitive words separately. Thus, it is difficult to compare the relative importance of these linguistic patterns with respect to psychological change. However, our results provide further support to the finding that writing about emotional upheavals may be essential to coping [30]. In addition, availability of others to provide support during expression of such emotional upheavals could result in a different mechanism in chat communication than found in disclosure writing interventions. For example, one study reported slightly increased use of affective words among participants within an Internet chat aftercare with increasing group size [33]. Further research will be helpful in understanding the association between the expression of positive and negative emotion words, and the improvements in eating-related cognitions during chat interactions.

One interesting finding was the linear association between improved emotional distress and longer duration of illness, i.e., those who had a more chronic illness course reported decreased emotional distress at the end of the chat groups. One explanation might be that the chat communication reduced the sense of isolation of chronic patients. Moreover, the weekly moderated group chats might have provided continuity between treatment and everyday life for dealing with chronic behavioural patterns that contributed to the amelioration of experienced emotional distress over time. This is also supported by participants’ comments which evaluate the feeling of being proactive about their health as helpful during their participation in the online program [16]. Determining the salient aspects of chat communication that boost emotional well-being in chronic patients with eating disorders could enable further adaptations of the intervention. To this end, one study investigated the text-based group processes within an Internet chat aftercare and found that higher satisfaction with the chat sessions, higher frequency of being mentioned by the other members and the therapist, and higher relative activity in comparison to the other group members were associated with improvements in psychological well-being of patients with affective, neurotic, and personality disorders [8].

The associations between higher baseline BMI and improvements observed in eating disorder-related cognitions and emotional distress highlight the importance of BMI as a predictor of improved outcome and are in line with previous research. Jones and colleagues [34] found that BMI and motivation indirectly influenced the treatment outcome via the ability to complete the day therapy programme. Sly and Bamford [35] described that higher body weight at start of inpatient treatment was related to better outcome.

This study has several limitations. Due to the small sample size the statistical power is low. The high number of correlational analyses would require Bonferroni correction but this was not taken into consideration due to the same reason. The diversity of the previous treatment modalities might have had an effect on the results obtained.

The content of the chat scripts were defined based on qualitative approach. Computerized approaches to identify the themes of the chat scripts (e.g., meaning extraction method [36]) could have been considered. We used the NooJ linguistic software [22] to calculate the frequency of corpus-based dictionary words. Relying on the frequency of the words limited our ability to determine the context within which they appeared under each theme. For example, the preferred usage of eating-related words may be a sign of forming a subculture, as in the “pro-ana” websites [11].

Despite the limitations, our study is informative for further prospective investigations. More studies are needed to clarify other linguistic characteristics. For example, it would be interesting to analyze the verb tenses, which draws attention to time, from past to future—or first-person and second-person pronouns, which relate to the relationship quality [31]. Another interesting research area would be the analysis of the functional words [26]. These may have therapeutic consequences.

To sum up, the expression of family of origin words following treatment termination was a linguistic predictor of improvement during group chat communication of patients with bulimic symptomatology in the current study. Further research on the language use of patients with eating disorders is necessary to give insights with respect to the maintenance factors after treatment. This can facilitate development of effective strategies to address them through online and offline communication mediums.
